# Piezodynamic Eradication of Both Gram-Positive and Gram-Negative Bacteria by Using a Nanoparticle Embedded Polymeric Membrane

**DOI:** 10.3390/pharmaceutics15082155

**Published:** 2023-08-17

**Authors:** Chan Chen, Shubham Roy, Jingjing Wang, Xiafen Lu, Siyi Li, Hao Yang, Minggang Cheng, Bing Guo, Yuzhong Xu

**Affiliations:** 1 Department of Clinical Laboratory, Shenzhen Baoan Hospital, The Second Affiliated Hospital of Shenzhen University, Shenzhen 518000, China; cx911108@163.com (C.C.); lxf153448817@163.com (X.L.); 13048800990@163.com (S.L.); yanghao93317@163.com (H.Y.); 2 School of Science, Shenzhen Key Laboratory of Advanced Functional Carbon Materials Research and Comprehensive Application, Harbin Institute of Technology, Shenzhen 518055, China; shubham.royju@gmail.com (S.R.); 13733188143@163.com (J.W.); 3 Shenzhen Key Laboratory of Flexible Printed Electronics Technology, Harbin Institute of Technology, Shenzhen 518055, China

**Keywords:** piezodynamic therapy, antibacterial therapy, PVDF-HFP, bacteria

## Abstract

Nowadays, bacterial infection is regarded as a serious threat to humankind, which needs to be taken care of. The emergence of antibiotic resistance and multidrug resistance (MDR) is rendering this situation more troublesome. However, several alternative treatment regimens have aided such diseases quite well in the recent past, among which dynamic antibacterial therapies combat this situation quite well. Among various dynamic therapies, piezodynamic therapy is a very recent avenue, in which mechanical stimuli have been exploited to treat bacterial infections. Herein, piezo-active bismuth ferrite-loaded poly(vinylidene fluoride-co-hexafluoropropylene) polymer has been utilized to eradicate gram-positive bacteria (*E. faecalis*) and gram-negative bacteria (*E. coli*). The sample has been designed in a free-standing membrane form, which, under soft ultrasound (~10 kHz), generates reactive radicals to ablate bacteria. Initially, the structure and morphology of the membrane have been substantiated by using X-ray diffraction and scanning electron microscopy methods; besides, Fourier transform infrared spectrum of the sample depicts a tremendously high value of polarizability and further confirms the piezo-activity of the membrane. More than 99% of *E. coli* and *E. faecalis* have been successfully eradicated within 30 min of ultrasound. Moreover, the solid-state structure and hydrophobic nature of the membrane help us to reuse it in a cyclic manner, which is possibly reported herein for the very first time. This novel membrane could be deployed in healthcare systems and pigment industries and could be exploited as a self-cleaning material.

## 1. Introduction

Bacterial eradication is a serious concern nowadays due to bacteria’s escalating and empowering potential against conventional antibiotics [1]. A pathogenic infection could even lead to death if improperly treated [2]. It is estimated that by the end of 2050 over 10 million lives each year could be lost due to the burgeoning rise of bacterial contaminants [3,4]. Overuse and misuse of antibiotics are leading us toward an even more complicated situation [5]. The rise of bacterial biofilms and antibiotic-resistant strains are some of the results of the horrible mismanagement of antibiotics [6]. In the recent past, nosocomial infection, predominantly, disrupted our living standards just after the COVID-19 crisis. Henceforth, new-generation technologies are required to combat the crisis [7].

In this regard, nanomaterials have become successful in delivering alternative therapeutics to ablate pathogenic infections [8]. Nanomaterials have excellent surface properties and are, therefore, capable of responding to different internal and external stimuli [9]. Moreover, stimulus-based nanomaterials show promise in the precise and rapid treatment of diseases. Several stimulus-based nanosystems have now been deployed in clinics. These stimuli can be broadly classified into two categories, namely, intrinsic stimuli, such as pH, inflammation microenvironment, etc., and external stimuli, such as light, heat, mechanical stress, etc. [1]. It is evident from the previous literature that stimulus-based therapeutics render better control over the treatment paradigm and with lower toxicity; besides, some of these stimulus-based nanotherapeutics can be guided and controlled in real time and, thus, open new opportunities in theranostic paradigms [10].

Piezo is a kind of stimulus which is generated by mechanical vibration and offers unlimited advantages in different sectors of materials science [11]. In reality, piezo-responsive materials convert mechanical energy into electrical energy, which has already been exploited by many groups to create nanogenerators, energy harvesters, etc. [12,13]. Very recently, some groups used piezo-stimulus to decontaminate wastewater from organic dyes and pharmaceutical pollutants [14,15]. Such decontamination uses the reactive radicals-producing ability of the piezo-responsive material, which in the presence of organic dye can eliminate contaminants in almost no time. Additionally, piezodynamic therapy offers deep penetration into the cells with excellent control, which was well established by Das and his group [16]. However, very little research has been conducted into the use of piezodynamic therapy in the domain of microbiology.

Disinfection of wastewater to minimize the risks associated with public health for exposure to pathogenic micro-organisms has become an essential process gathering huge attention. Every year, millions of infections are reported, arising from the consumption or utilization of contaminated water [17]. Since it is currently a matter of concern requiring an immediate solution, various types of research have been devoted to the development of advanced processes to inactivate or kill such bacteria. This work demonstrates a facile approach to inhibiting pathogenic gram-positive and gram-negative bacteria by virtue of mechanical stress. Herein, *Escherichia coli* (*E. coli*) and *Enterococcus faecalis* (*E. faecalis*) were selected as representatives of gram-negative and gram-positive bacteria, respectively. Both bacteria are regarded as coliform bacteria that can act as indicator organisms to represent a broad spectrum of human pathogens found in effluent, sewage, or wastewater having high tolerances or resistance properties which make them challenging to handle [18].

**Scheme 1:**
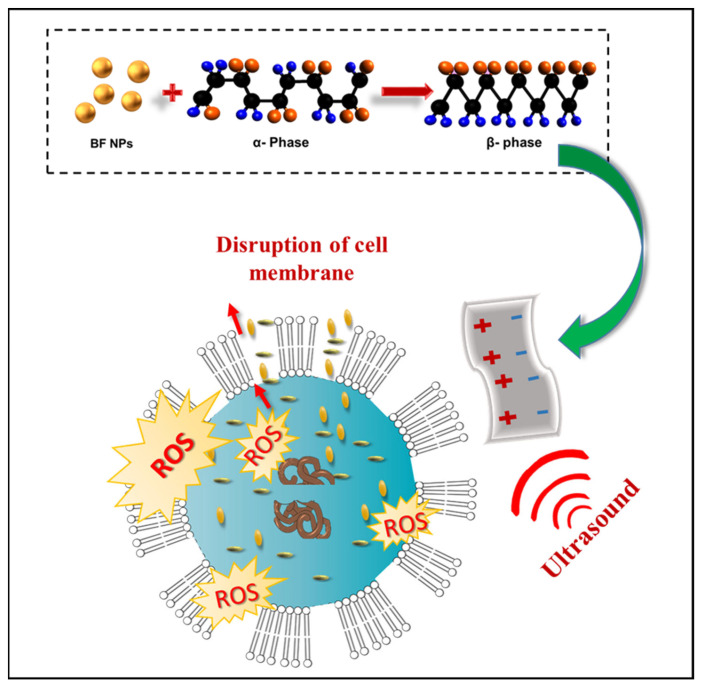
ROS-mediated bacterial inhibition mechanism depicting membrane lysis and leakage of cytosol.

In the present study, we deploy bismuth ferrite (BF), which is known to have excellent ferroelectric properties with poly(vinylidene fluoride-co-hexafluoropropylene) (PVDF-HFP) to ablate the bacterial infection. Both BF and PVDF-HFP are reportedly piezo-active. However, their mechanisms of piezo-polarization are entirely different to each other. On the one hand, BF offers polarization through its structural bending, which separates the electron and holes to either side of the sample and creates a charge-center separation under mechanical stress. On the other hand, PVDF-HFP is known to have different conformations (or phases), namely, α, β, γ, and δ. Among these, β-phase offers the highest polarity owing to its all-trans conformation (TTTT). In this regard, the synergistic contribution of BF and PVDF-HFP in the domain of polarization-driven piezo-activity has been rigorously investigated. Different characterization techniques, such as X-ray diffraction (XRD), field emission scanning electron microscopy (FESEM), transmission electron microscopy (TEM), and Fourier transform infrared spectroscopy (FTIR), have been utilized to substantiate the purity, microstructure, morphology, and overall polarizability of the as-prepared membrane samples. These solvothermally synthesized BF nanoparticles enhance the polarizability of the PVDF-HFP membrane and are found to have a superior piezo-responsive nature. Under soft ultrasound (10 kHz), this BF-incorporated PVDF-HFP membrane exhibits more than 99% killing of *E. coli* and *E. faecalis* in only 30 min (Scheme 1). Moreover, the reusability of the membrane has been checked by recycling it for, at least, three consecutive cycles for bacterial therapy. The membrane exhibits superior reusability under such conditions and offers almost ~98% bacterial inactivation in its third cycle in both cases, making it a revolutionary weapon against the superbugs in the coming days.

## 2. Materials and Methods

### 2.1. Materials

Nitrate salts of bismuth (Bi(NO_3_)_3_·5H_2_O) and iron (Fe(NO_3_)_3_·9H_2_O) along with solvents like *N*, *N*-Dimethylformamide, acetone (HPLC grade), and ethanol (HPLC grade) were purchased from Merck. Additionally, citric acid, ethylene glycol, and 2-methoxy ethanol were purchased from Sigma-Aldrich. All the purchased reagents were of analytical grades and were used without further purification. In order to synthesize polymeric membranes, Poly(vinylidene fluoride-*co*-hexafluoropropylene) was procured from Sigma-Aldrich. The deionized water was used throughout the experiment with a resistivity of, at least, 18.2 MΩ-cm. All the glassware used in our experiments was cleaned with aqua regia solution followed by rinsing with ultrapure water.

### 2.2. Characterizations

The zeta potential was measured to predict the surface charge of the BF nanoparticles. In a typical experiment, 1 mg of BF nanopowder was dispersed in 2 mL of DMF and treated with ultrasound. After 60 min of ultrasound, a clear suspension was formed, which was then collected in the cuvette system and placed in a Zetasizer (from Malvern) for obtaining the zeta potential value.

The XRD diffractograms were collected by using a D-8 Advance, Bruker AXS, Wisconsin, USA, diffractometer equipped with a Cu-Kα (1.5418 Å) target to obtain the structural information. The scan speeds in each case were set at 3 s/step with an accelerating voltage of 35 kV and a current of 35 mA.

In order to obtain the FESEM images, an F-50, FEI scanning electron microscope was utilized to observe the morphological features of the synthesized nanocomposites. Typically, powdered samples were placed over carbon grids and then coated with gold prior to the microscopy; polymeric membranes were cut into tiny pieces and placed over the carbon grids before the gold deposition. The samples were then placed under an accelerating voltage, ranging between 5 kV and 20 kV, under a high vacuum environment for recording the micrographs.

For the TEM micrograph of the BF nanopowder, a JEM-2100 Plus JEOL transmission electron microscope was employed with an accelerating voltage of nearly 200 kV. The BF nanopowder was initially ground for a prolonged period and dispersed in acetone prior to the ultrasonic treatment. Subsequent ultrasound reduced the agglomeration and released any impurities. A tiny drop of the ultrasound-treated sample was then cast over a 300-mesh carbon-coated copper TEM grid and dried in a vacuum prior to microscopy.

The FTIR spectrometry was performed in a Shimadzu Infrared IR Affinity spectrometer in the wavenumber ranging between 400 and 1600 cm^−1^. The membranes were exposed directly to the laser beam and placed in the instrument’s designated sample holder.

### 2.3. Synthesis of the Bismuth Ferrite NPs

In a typical synthesis process, 0.002 M of bismuth and iron salts were dissolved in 15 mL of 2-methoxy-ethanol to obtain a homogeneous suspension. During the formation of this suspension, 0.002 M citric acid and ethylene glycol at a ratio of 1:1 were added while stirring for the formation of sol. Moreover, the ethylene glycol herein acts as the growth-controlling agent, which results in the formation of the particles in the nano regime. The stirring was continued for another 120 min at 60 °C and, thereby, the entire solution was transferred into a Teflon-lined stainless-steel autoclave for solvothermal treatment. The solvothermal treatment was performed for 20 h at 110 °C. After completion, the solid precipitates were collected from the bottom of the autoclave, washed several times, and dried. The dried precipitates were further heat-treated at 600 °C for 2 h in a muffle furnace, ground into fine powders, and marked as BF.

### 2.4. Preparation of the PVDF-HFP@BFO Composite

A facile solution casting method synthesized the PVDF-HFP@ bismuth ferrite nanocomposite membranes. Precisely, 0.5 g of PVDF-HFP was dissolved in 10 mL of DMF under vigorous stirring at 70 °C. The BF nanoparticles were then added at different weight percentages (5 and 15%). After overnight stirring, the suspensions were separately poured into glass Petri dishes and were placed in a hot air oven at 80 °C for another 6 h for drying. The dried membranes were peeled off the glassware and marked as BF@PV5 and BF@PV15 for 5 and 15% BF-doped samples, respectively. A pure PVDF-HFP membrane was also prepared for comparative studies and marked as BF@PV0.

### 2.5. Antibacterial Experiments

*Escherichia coli* or *E. coli* (DH5α, MTCC-1652) and *Enterococcus faecalis* or *E. faecalis* (MTCC-439) were selected as the model bacteria in the assay [19]. Sterile Luria–Bertani (LB) broth was inoculated with bacterial suspension of 0.5 McFarland standard prior to evaluation of piezodynamic inactivation performance of BFO@PVDF membrane [20]. The inoculated solutions were divided into two parts. The membrane (1 cm × 1 cm) was added to the inoculated solution and was subjected to ultrasonication for 30 min. Another part without a membrane was kept near the experimental sector and treated as a control. At an interval of 10 min, 1 mL aliquot was withdrawn from both parts and diluted 1000 times with sterile LB broth prior to uniform coating on solid LB agar plates. The plates were then maintained at 37 °C for 24 h and the colonies formed were counted. A similar protocol was followed for subsequent two more times using the same membrane to determine the reusability efficiency.

The amount of ROS generation during the experimental tenure was measured using 10 µM 2′, 7′-dichlorofluorescein diacetate (DCFH-DA) solution. The aliquots collected were washed twice (5 min each at 5000 rpm) with sterile phosphate buffer saline (PBS) solution and were added under darkness. After 30 min, the amount of fluorescent 2′, 7′-dichlorofluorescein (DCF) production was measured using a fluorescence spectrophotometer (BIOTEK) at an excitation wavelength of 490 nm and emission wavelength of 515 nm. Morphological alterations in the bacteria were studied from FESEM micrographs. Initial and final aliquots were washed twice with filtered PBS solution and 2.5% glutaraldehyde was added to each. After 2 h, they were dehydrated with ethanol at serial dilute, drop-casted on grease-free, clean coverslips, sputtered with gold after drying, and subjected to FESEM.

## 3. Results and Discussion

### 3.1. Design Rationale of the As-Prepared BF@PV Samples

**Figure 1:**
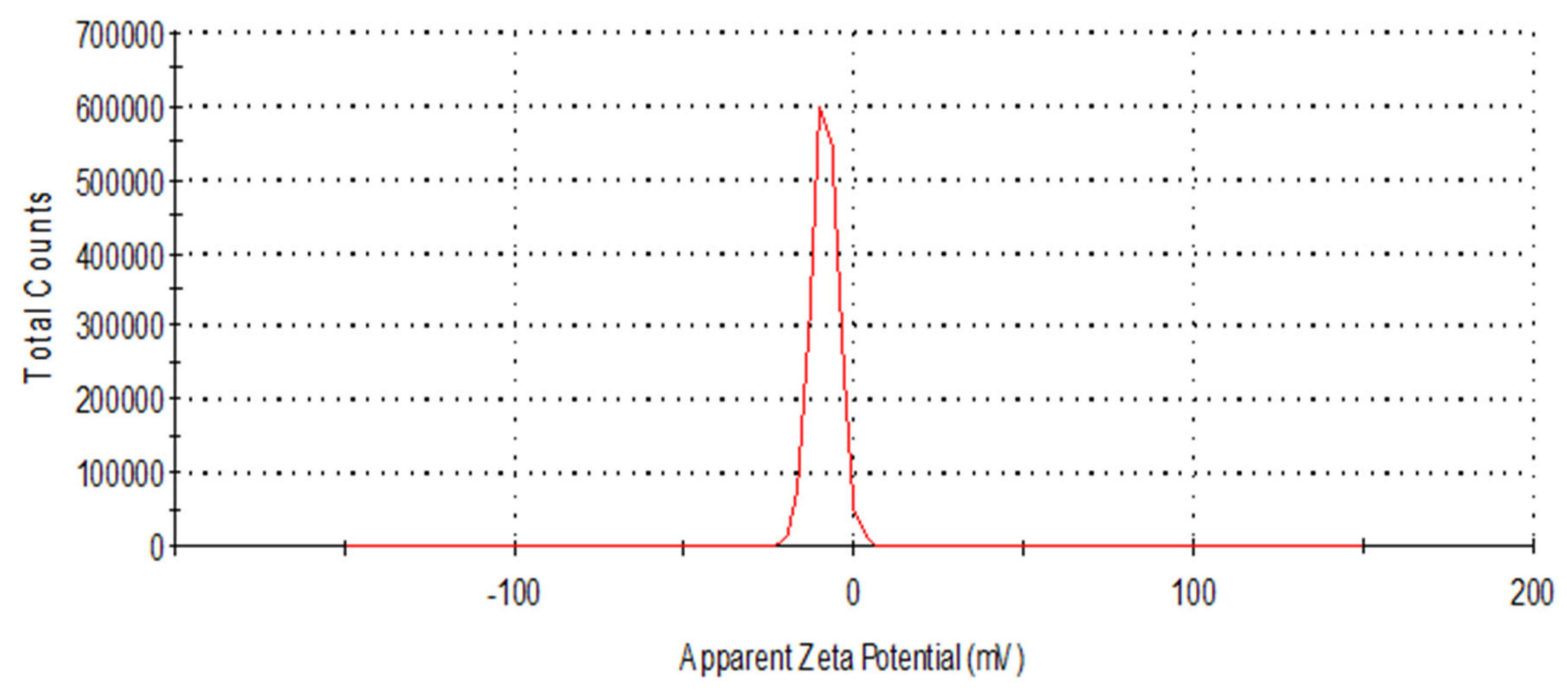
The apparent zeta potential of the BF nanoparticles displays a high negative surface charge.

Typically, BF nanoparticles have a negative surface charge [21]. In our case, it was found to be −8.21 mV (Figure 1). Such a high negative surface charge validates its stability in solvent and features a promising electronic interaction of negatively charged BF nanoparticles with the positive CH_2_ moiety of the PVDF-HFP polymer. Evidently, such electronic interaction disrupts the α-association of the PVDF-HFP, which normally has a TGTG confirmation and changes it into the polar β-phase [22]. In reality, this polar β-phase has an all-trans (TTTT) conformation, which is only possible if all hydrogen and fluorine atoms are polarized on either side [14].

**Figure 2:**
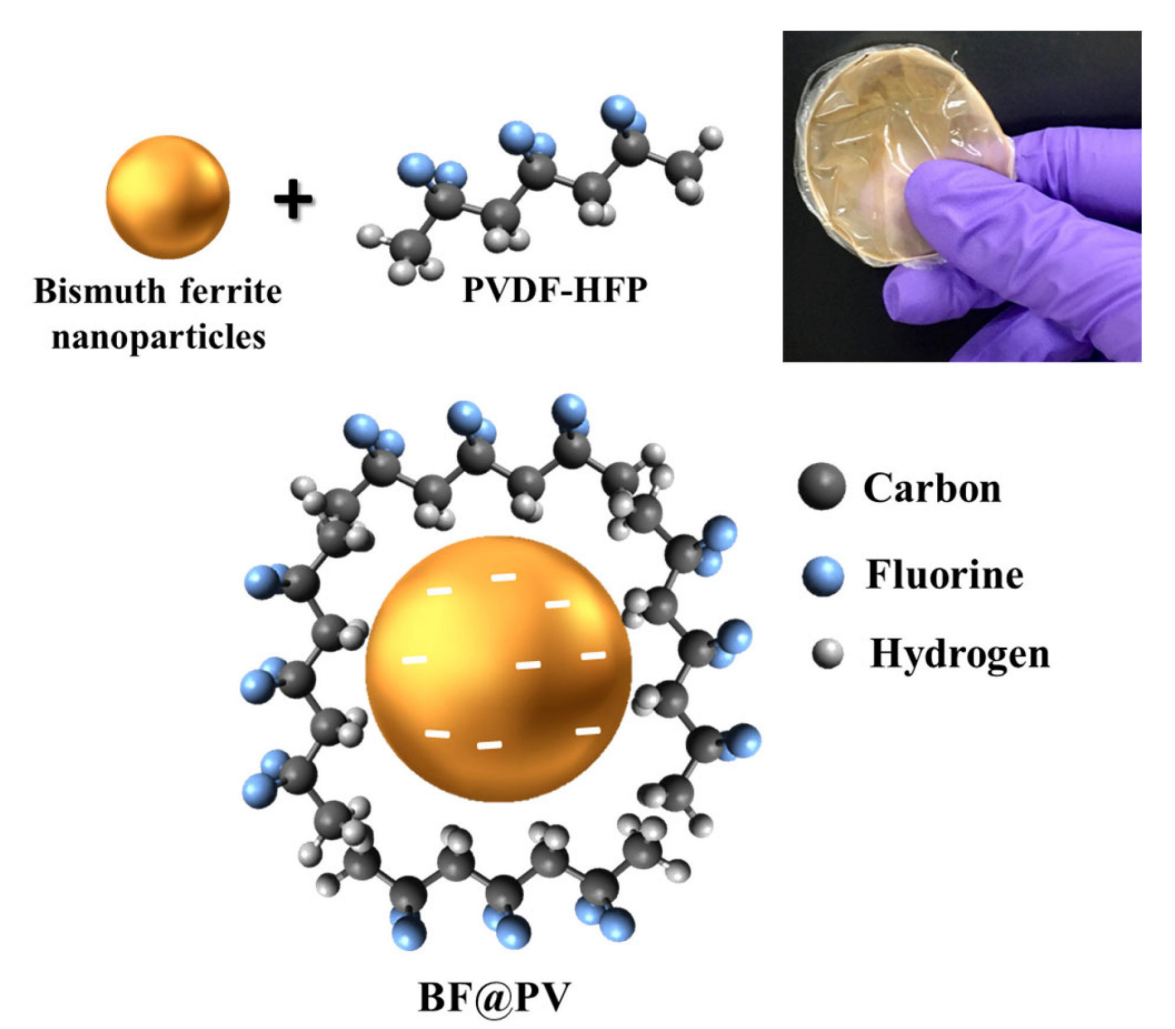
Plausible conformation of the BF@PV samples and a real-time photograph of the BF@PV5 membrane is in the inset.

Herein, the negative surface of BF nanoparticles attracts the CH_2_ moiety of the polymer and forms a TTTT conformation, which renders it sufficiently polarizable. Figure 2 depicts the overall molecular arrangement of the BF@PV nanocomposites and confirms a high polarity of the sample, which could be ascribed to their piezo-response.

### 3.2. Structural Characteristics of the Synthesized BF@PV

**Figure 3:**
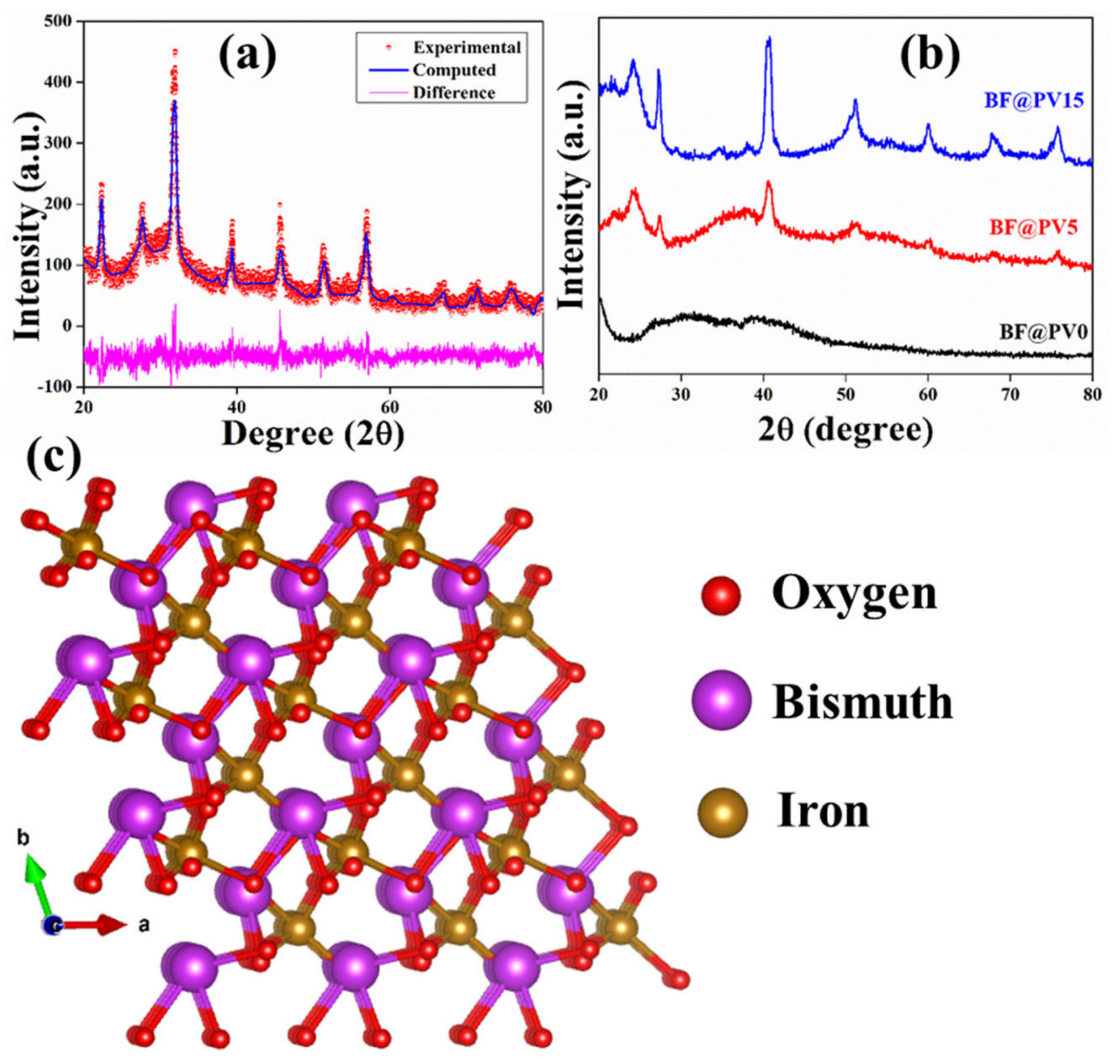
(**a**) XRD patterns of the as-synthesized BF nanoparticles, (**b**) X-ray diffractograms of the BF nanoparticles incorporated PVDF-HFP membranes at different BF-concentrations, where BF@PV0 represents pristine polymer, BF@PV5, and BF@PV15 represents 5 and 15 wt% loaded BF in the polymeric matrices respectively, and (**c**) the microstructure of the prepared BF nanoparticles.

The X-ray diffraction method was applied to initially asses the purity and microstructure of the as-synthesized BF samples and, thereby, to study their incorporation into the PVDF-HFP matrix. The XRD pattern of the BF nanopowder was determined to substantiate its purity and successful formation, and, thereby, the XRD patterns of the membranes were determined (Figure 3).

It was observed that the characteristic diffraction maxima at 2θ = 31.98° corresponding (110) plane corresponds with pure bismuth ferrite which suggested that the rhombohedral crystal structure of BF with a space group R3c at room temperature (as stated in the JCPDS card 86-1518) are entirely analogous with the obtained XRD pattern [21]. The absence of any undesired diffraction peak rather suggests the phase purity of the synthesized BF nanostructures. The XRD patterns were further refined by using the standard Rietveld refinement technique. For this, MAUD v2.94 software was used for superposing the diffractogram of the standard BF sample over the experimental pattern to collect the crystallographic information of the synthesized BF sample. Such information is required to study the interaction of BF and PVDF-HFP polymer. Initially, the background points were refined and, subsequently, the instrumental broadening, peak intensity, and Gaussian broadening of the diffraction maxima were refined. The microstructural parameters, such as unit cell parameters and cell volume, were refined accordingly. After 20 cycles of the refinement of each parameter, the global reliability parameters were seen to be satisfactory (R_wp_ = 13.4%) and the unit cells were visualized utilizing VESTA v3.5.2 software (Figure 3c). It was observed that the R3c space group had been formed in the BF microstructure, which substantiates more polarity in the BF phase. Moreover, its perovskite-like structure resembled the previous studies, which validates the successful synthesis of the BF nanoparticles [21]. The microstructure and other parameters are depicted in Table 1.

Furthermore, the BF-incorporated PVDF membranes offer semi-crystalline XRD patterns. Several distinct diffraction maxima were found at 17.7° (100), 18.2° (020), 39.3° (211), and 20.3° (110), which corresponds to α, α and γ, γ, and β-phases of PVDF-HFP polymer, respectively [23]. The striking enhancement of the polar β-phase compared to that of α and γ phases in the BF@PV5 suggests that the polarity of this sample attains maximum value at that specific composition.

### 3.3. Morphological Features of the BF@PV Nanocomposites

**Figure 4:**
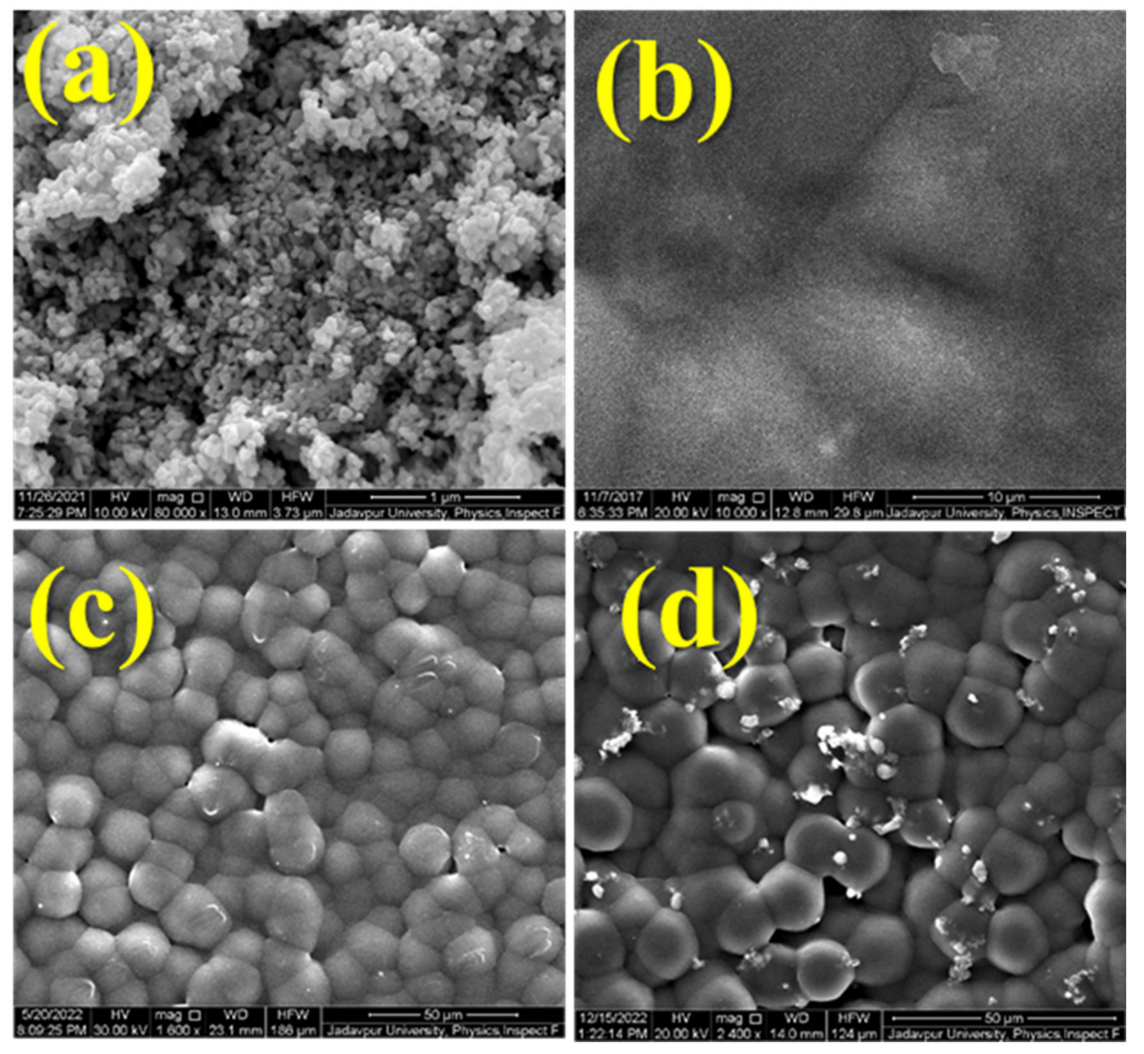
FESEM micrographs of the synthesized (**a**) BF NPs and (**b**–**d**) the synthesized membranes showing the microstructures of pure PVDF (BF@PV0), 5% BF in PVDF matrix (BF@PV5), and 15% BF in PVDF matrix (BF@PV15), respectively.

**Figure 5:**
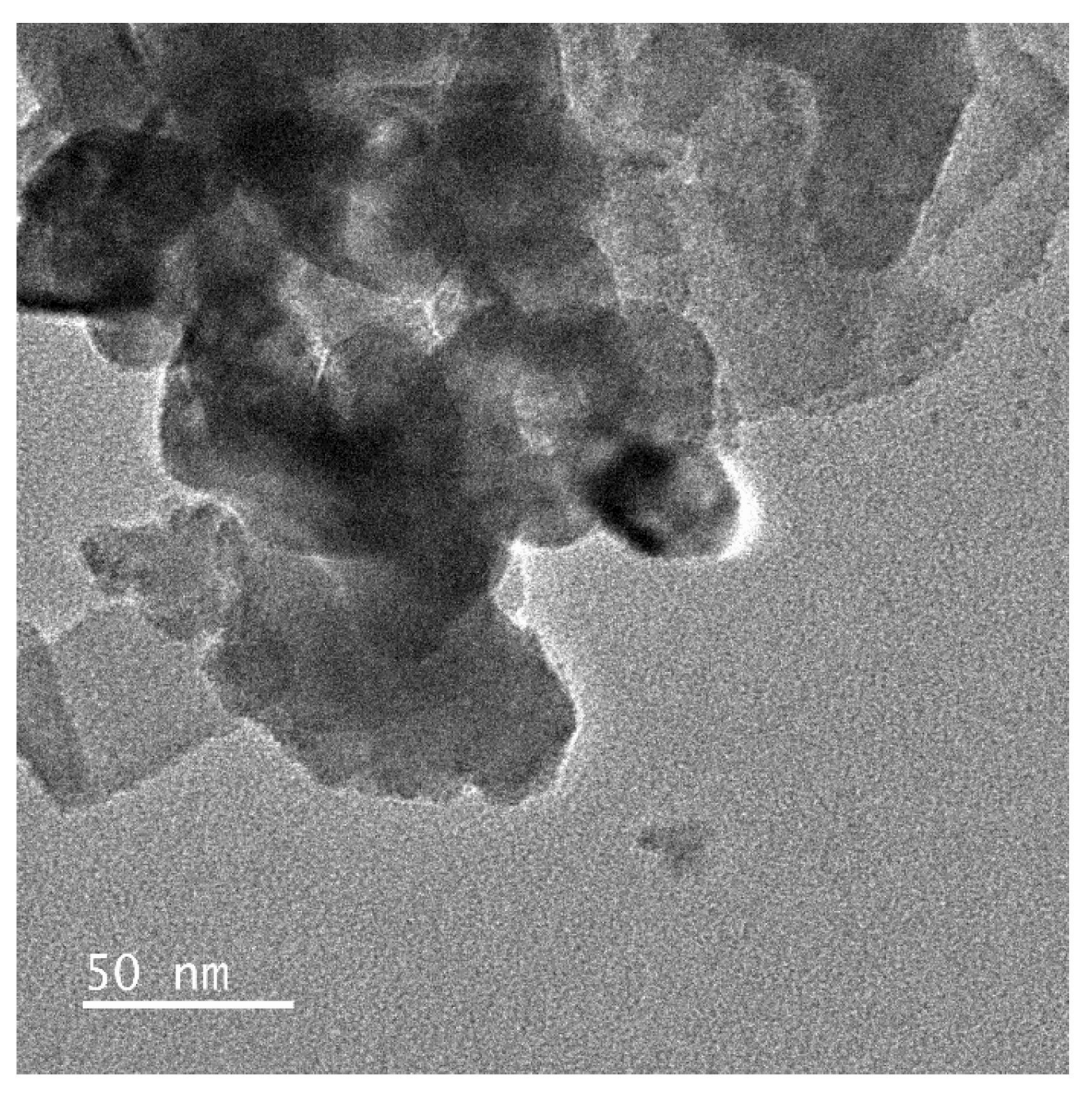
TEM micrograph of the solvothermally synthesized BF nanoparticles.

Field emission scanning electron microscopy (FESEM) and transmission electron microscopy (TEM) are efficient characterization tools for obtaining information on the structure and morphology of a material (Figure 4 and Figure 5), respectively.

It is evident from the FESEM images that the incorporation of the BF-nanoparticles into the PVDF-HFP matrix drastically alters the morphology of the samples. Notably, the surface roughness is enhanced and the formation of ‘globules’ has taken place with increasing doping concentration (Figure 4). The formation of such globules suggests the successful incorporation of the dopant, i.e., BF nanoparticles, into the polymeric matrix of the PVDF-HFP and, thereby, substantiating the amalgamation [24].

Additionally, the TEM micrograph of the BF nanoparticles was obtained to validate its size and shape. Figure 5 depicts the TEM image of the BF nanopowder showing the irregular globular morphology of the nanoparticles. Moreover, some agglomeration can be seen in the image, which may result due to the smaller size of the particles (30–50 nm, in this case). In reality, smaller particles exhibit enhanced surface area and, therefore, tend to form agglomerates with neighboring particles [25]. However, the incorporation of the BF-nanoparticles into the polymeric matrix of PVDF-HFP restricts this trend and could be useful in real-life applications.

### 3.4. Estimation of the Degree of Polarization

**Figure 6:**
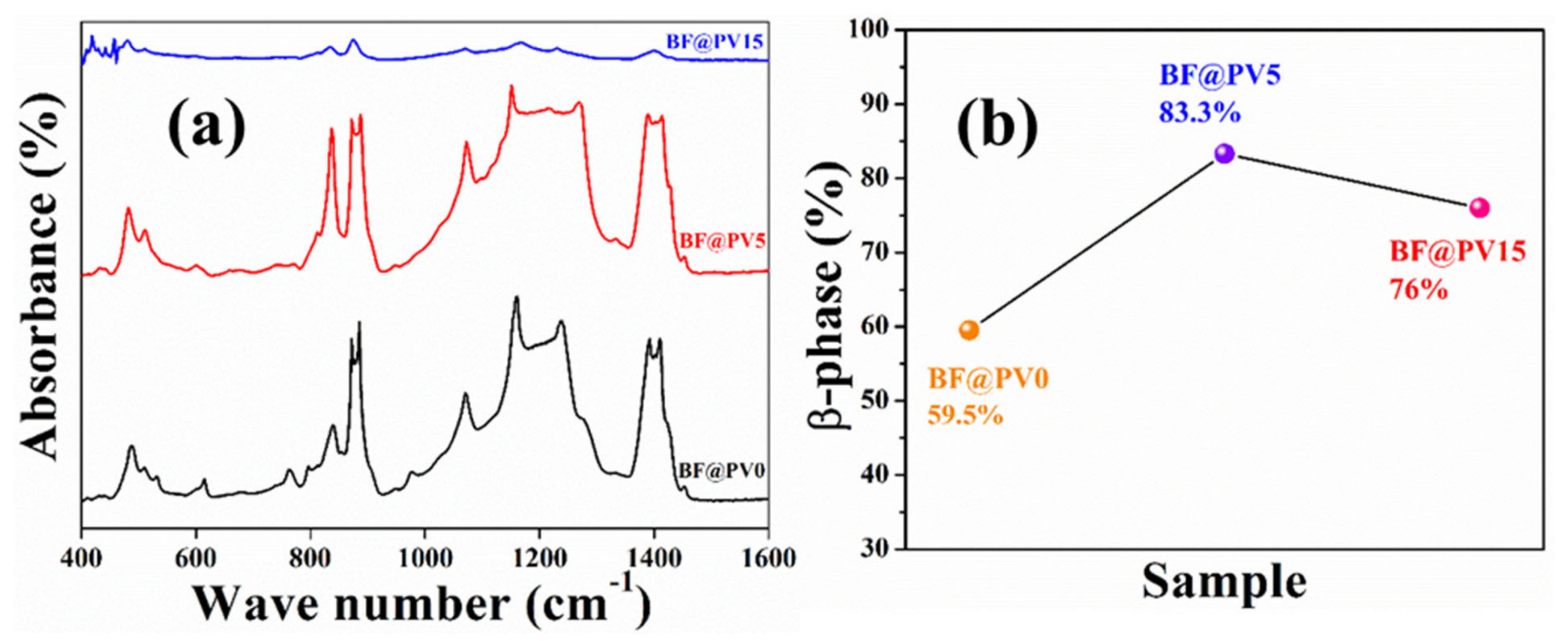
(**a**) FTIR spectra of the synthesized membrane samples and (**b**) corresponding β-phase formation in percentage scale showing highest polarity in BF@PV5 sample.

In order to estimate the degree of electronic polarization, which is primarily responsible for the piezo-responsiveness of the membrane samples, we exploited several methods. Initially, we started exploring the polarizability of the PVDF-HFP matrix. In reality, PVDF-HFP has a CF_2_-CH_2_ trans-gauche (TGTG) conformation, which, upon doping with certain elements, converted into all-trans (TTTT) polar conformation [26]. On the one hand, the TGTG conformation is known to have a lower polarization and crystalline index (α-phase). On the other hand, TTTT conformation is known as β-phase, having superior polarizability and piezo-response [26,27]. The characteristic α and β-peaks of PVDF can be found in a Fourier transform infrared spectrum (FTIR), which we employed to estimate the polarizability and the amounts of β-phase formation upon BF-doping (Figure 6).

It was found that the absorbance of the β-peaks located at 840 cm^−1^ owing to the CH_2_ rocking, CF_2_ stretching, and skeletal C-C stretching had been enhanced compared to the corresponding α-peaks formed due to the skeletal bending of CF_2_ [28]. Otherwise, all the absorbance bands are well in accordance with the previously reported values. The percentage of β-phase formation in each case was estimated by using the Lambert-Beer law as mentioned below,

(1) $$F\left(\beta \right)=\frac{A\left(\beta \right)}{\frac{k\left(\beta \right)}{k(\alpha )}*A\left(\alpha \right)+A(\beta )}$$

where A(α) and A(β) are the absorbance bands for the α phase at 764 cm^−1^ and β phase at 840 cm^−1^, respectively. K(α) (6.1 × 10^4^ cm^2^ mol^−1^) and K(β) (7.7 × 10^4^ cm^2^ mol^−1^) are the absorption coefficients at the respective wavenumber [29,30].

The calculated β-phase fraction is highest in BF@PV5, which is approximately 83.3%, whereas, BF@PV15 has comparatively lower polarizability (76%). Such a lowering of polarization may be attributed to the over-doping of the BF-nanoparticles, which might result in disruption in the bonding networks. BF@PV0, on the other hand, possesses 59.5% of β-phase. Hence, it is evident that BF@PV5 could be used as a piezoelectric material for further applications and, thereby, used herein for all antibacterial studies.

### 3.5. Mechanism of the Piezo Response

If we closely look at the mechanism of the piezo-responsive nature of the membrane (BF@PV5), we may find some interesting features of the sample. Precisely, under mechanical stress, the self-poled membrane activates its positive and negative charges, which migrate towards two different sides creating a charge center separation. External mechanical stimulus drives these charges toward the surface of the membrane. In the presence of any polar solvent, such as water, these charges produce reactive oxygen species (ROS) by reacting with negative hydroxyl ions present in the solution [11].

Here, the steps of ROS production are highlighted briefly:

$$BF@PV5+mechanicalenergy\to BF@PV5(e^{-}+h^{+})$$

Anodic reactions:

$$4e^{-}+4H_{2}O\to 4OH^{-}+4H^{*}$$

$$4H^{*}\to 2H_{2}$$

Cathodic reactions:

$$4OH^{-}\to 4e^{-}+4OH^{*}$$

$$2\left({OH}^{*}+{OH}^{*}\right)\to 2H_{2}O+2O^{*}$$

$$2O^{*}\to O_{2}$$

Such ROS production is highly desired in several applications, such as pollutant degradation and bacterial ablation, etc.

### 3.6. Piezodynamic Antibacterial Studies

**Figure 7:**
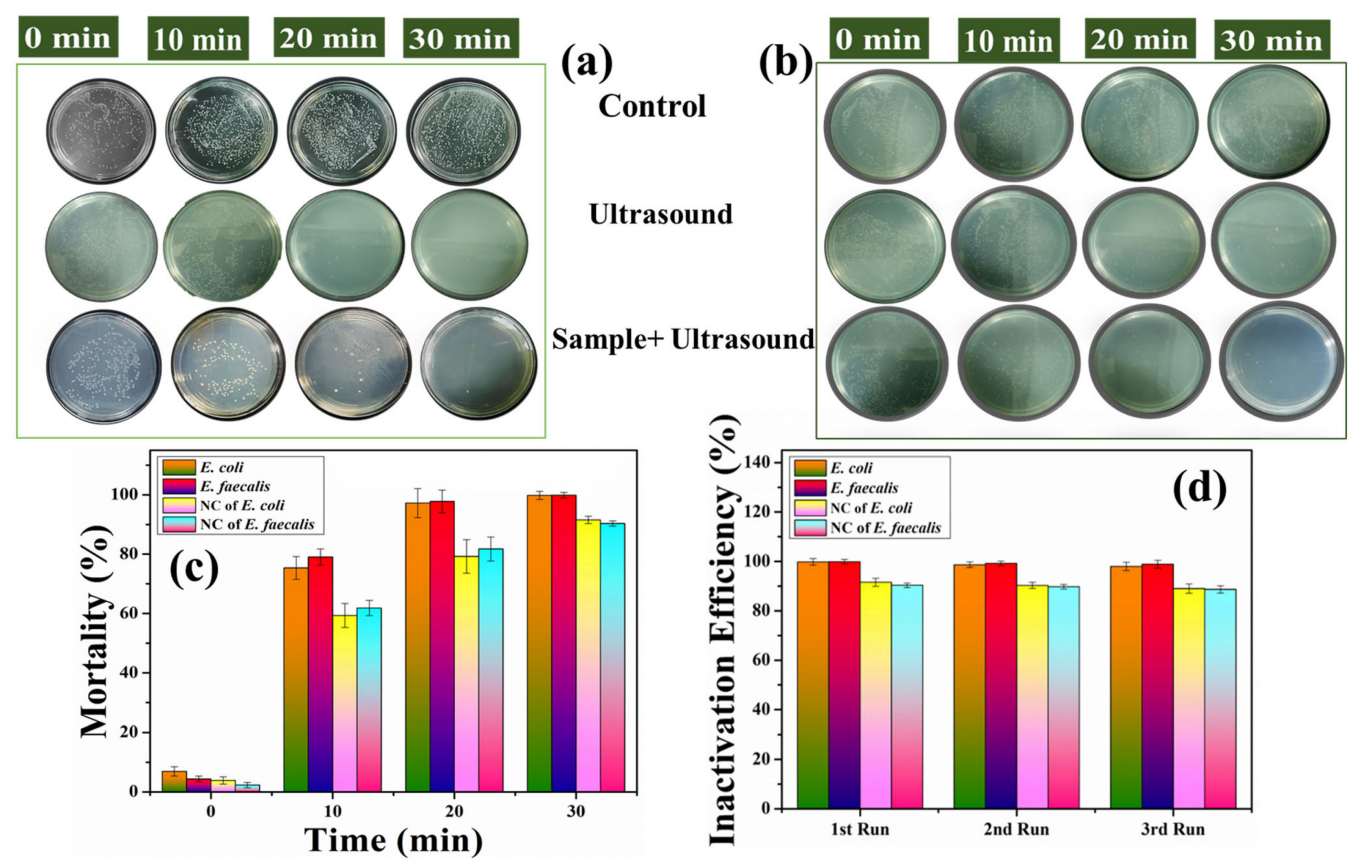
Photographs of surviving colonies of (**a**) *E. coli* and (**b**) *E. faecalis* in the solid agar plates observed after 24 h of incubation; (**c**) mortality percentage of *E. coli* and *E. faecalis*; and (**d**) analysis of disinfection efficacy of BF@PV membrane for three cycles to understand the reusability efficiency.

**Figure 8:**
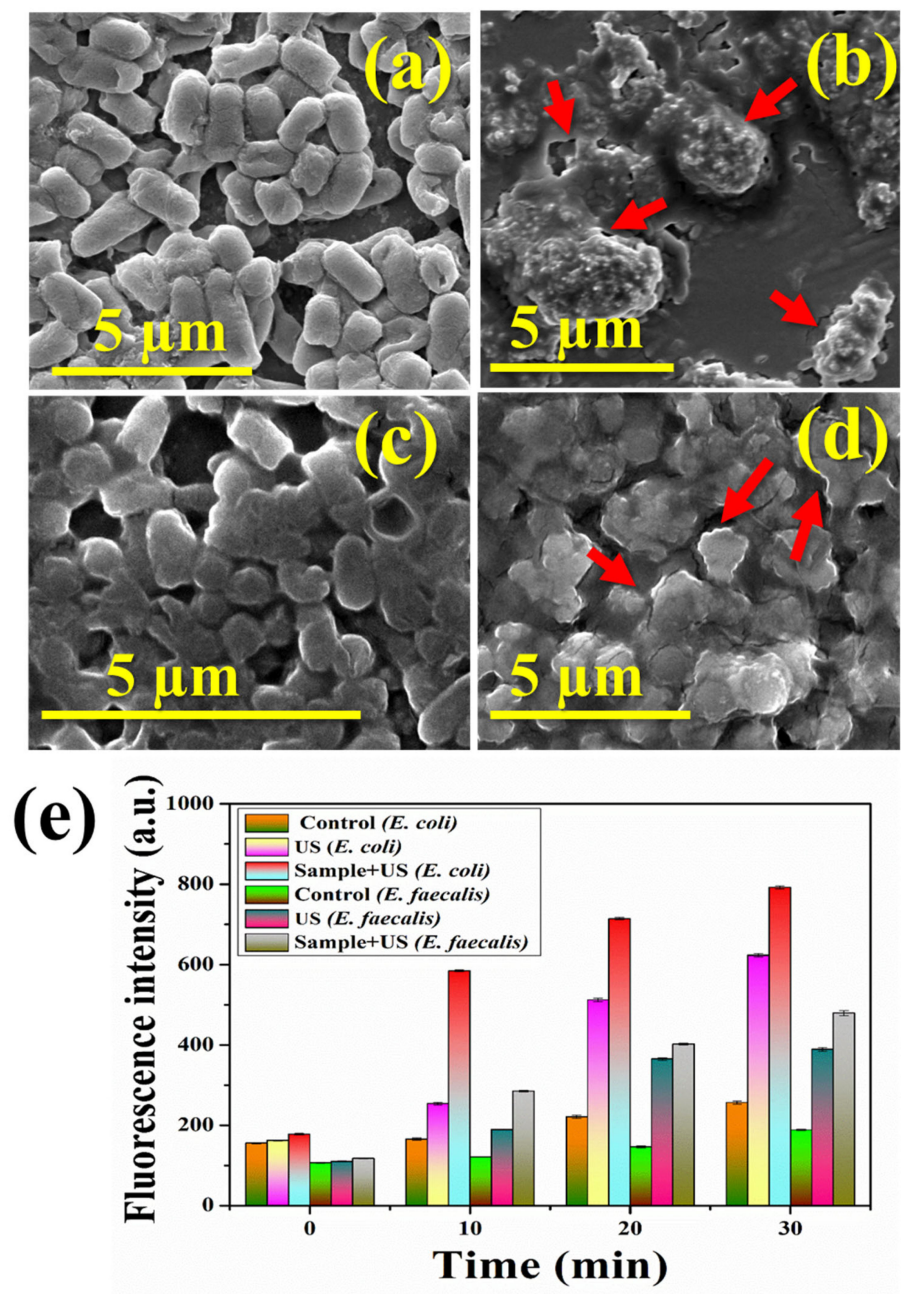
FESEM micrographs for (**a**) control of *E. coli* and (**c**) *E. faecalis*, illustrating smooth and intact cell membrane and subsequent loss of membrane integrity and membrane disruption in *E. coli* (**b**) and *E. faecalis* (**d**) due to piezodynamics in the presence of BF@PV under ultrasonic vibrations—the red arrows indicate the leakage of intracellular components occurring due to piezodynamics; and (**e**) determination of ROS generation from the fluorescence intensity.

The piezodynamic inactivation of viable *E. coli* DH5α and *E. faecalis* cells by BF@PV membrane under ultrasonication has been studied at time points 0, 10, 20, and 30 min and the disinfection effect results are illustrated in Figure 7. Figure 8a,b portrays the efficient reduction in the number of viable colonies of *E. coli* and *E. faecalis,* respectively, on the solidified agar plates due to the piezodynamic degradation of the bacterial cells. To determine the antibacterial effect, percentage mortality was calculated from the colonies grown on the agar plates using the following equation [31]:

(2) $$M\left(\%\right)=\frac{N_{B}-N_{S}}{N_{B}}\times 100$$

where, M, N_B_, and Ns represent the percentage mortality, and the average number of colonies in the control and treated plates, respectively. A significant enhancement in the mortality rate of 75.35, 97.20, and 99.79% was observed at 10, 20, and 30 min, respectively, in the case of the gram-negative *E. coli*, while a higher mortality rate of 79.03, 97.76, and 99.87% was observed at the same time interval in gram-positive *E. faecalis* (Figure 7c), indicating effective disinfection. This efficiency remains unhindered even after repetitive use of the membrane, as observed from the disinfection cycle assay for three runs (Figure 7d). The FESEM micrographs (Figure 7a–d) confirmed the distortion in the bacterial membrane due to mechanical stress generated from the piezodynamic phenomenon. Simultaneously, a significant enhancement of fluorescence intensity was observed (Figure 8e), suggesting a high level of production of DCF, indicating high ROS generation.

Piezodynamic degradation of bacterial cells using generally acoustic waves involves thermal, chemical, and mechanical stress [32]. The liquid medium in contact with the ultrasonic waves forms tiny bubbles at the end of wave propagation, and these bubbles travel randomly, rise up, and, ultimately, disappear on the surface. When the acoustic waves pass through the culture medium, high pressure is usually induced on the piezo-responsive material due to cavitations in the medium as the bubbles collapse [33]. Huge energy is released in the medium as the bubbles collapse, which is high enough to displace atoms, resulting in internal polarization. This may further generate ROS like OH• which can induce bacterial disintegration [11]. Moreover, the catalase enzymes and bacterial antioxidant defense system have an inherent tendency to resist external and intercellular damage. When they are exposed continuously to the mechanical stress arising from the acoustic waves, ROS generation such as OH•, O_2_^−^, H_2_O_2_, h^+^, and e^-^ increases, which incapacitates the defense capacity of the bacteria. Such oxidative or free radicals at first disrupt the protective cell wall and target the cell envelope, causing dents, wrinkling, and perforations in the cell membrane [32]. The negatively charged bacterial surface further facilities enhancement of membrane permeability as the positively charged ions, such as h^+^ in the bacterial environment generates electrostatic forces with the negatively charged lipid bilayer. This impairs the normal functioning of the metabolic electron chain, thus disrupting the trans-membrane electron transfer. Furthermore, the free radicals oxidize the lipid proteins in the membrane [34]. Lipid peroxidation and impaired cell permeability allow penetration of the radicals in the bacterial cell and nucleus, which results in oxidative damage to the intracellular organelles and nuclear components. Oxidative damage to cell proteins, enzymes, DNA, and RNA inhibits normal physiological functioning and the newly torn membrane causes leakage of internal cytoplasmic materials. This was confirmed by the FESEM study (Figure 8) as the irreversible membrane damage and subsequent bacterial rupture due to enhanced permeability, releasing internal materials due to the action of piezodynamics and amplified pressure generation in the BF@PV membrane. Such leakage of cytosol from the otherwise smooth and intact bacterial cells ultimately results in bacterial death.

The Fe^3+^ ions in the BF@PV5 structure can initiate electrostatic interactions with the negatively charged bacterial membrane, leading to the oxidation of surface molecules and subsequent initiation of bio-destructive effects in the bacteria. Moreover, the polarization ability uplifts the production of ROS, facilitating the degradation of the membrane. As evident from the plate-counting method and evaluation of ROS generation, the gram-negative *E. coli* shows greater tolerance to the piezocatalysis compared to *E. faecalis*. *E. coli* is known to have one of the fastest multiplication rates compared to other bacterial strains [35]. Moreover, *E. coli* being a gram-negative bacterium contains a fairly complex outer membrane, having an additional outer lipopolysaccharide membrane that provides a strength of their structural integrity [36]. Thus, it provides a slightly higher resistance to membrane damage than *E. faecalis*, which results in comparatively lower inactivation and disinfection rate in the gram-negative *E. coli.*

The reactive species combine with DCFH-DA, forming a high amount of fluorescent DCF, as evident from the ROS analysis. Thus, the drastic reduction in the viable bacterial colonies is in accordance with the ROS and FESEM data. Furthermore, the recyclability assay shows insignificant alteration in the bacterial degradation efficiency in the three cycles performed, thus confirming the commercial applicability of the membrane. Hence, it was confirmed that the BF@PV membrane can successfully eliminate coliform/pathogenic bacteria under exposure to mechanical stress, such as acoustic energy within a span of 30 min by virtue of inherent piezodynamic properties.

Herein, we compared our results with previous works on piezodynamic antibacterial therapies and listed them in Table 2. The comparative analysis shows the remarkable efficacy of the synthesized BF@PV5 sample in eradicating *E. coli* and *E. faecalis*, paving the path toward possible clinical translation.

## 4. Conclusions

In summary, bismuth ferrite nanoparticles have been successfully synthesized and incorporated into the polymeric matrix of PVDF-HFP at different weight percentages to obtain an optimized doping concentration. Various characterization tools such as XRD, TEM, and FESEM were utilized to obtain the structure and morphological features of the sample, whereas FTIR spectra helped us in revealing the polarizabilities, which further confirms the piezo-activity of the samples. Notably, the 5% BF-loaded sample (BF@PV5) depicted the highest piezoelectric nature and was thereby used in piezodynamic antibacterial therapy against pathogenic *E. coli* and *E. faecalis* bacteria. It was observed that almost 99% of both bacteria have been eradicated under the low-energy ultrasound treatment in the presence of the BF@PV5 sample. Higher inactivation of bacterial cells was observed for the gram-positive *E. faecalis* than the gram-negative *E. coli* cells. Further experimentations lead us to the generation of excessive ROS in bacterial cells, which acted as a lethal weapon against bacterial contamination. Moreover, the piezo-active membrane depicted a self-sustaining feature with superior reusability. It was found that a single membrane could be used several times to treat bacterial contamination, which not only makes it reusable but also cost-effective.

Piezodynamic inactivation of pathogenic bacteria has been developed very recently; thus, there is a huge scope for exploring new materials and new multimodal technologies in this domain. Similar to any other dynamic therapy, piezodynamic therapy also invades bacteria by ROS formation. However, its ability to ablate deep-seated infection may offer new avenues in the near future. Moreover, piezodynamic therapy could arrest antibiotic-resistant strains and other superbugs, which will definitely bring new ideas to improve its efficiency and overall therapeutic paradigms.

## Figures and Tables

**Table 1:** Microstructural representation of the BF nanoparticles.

Parameters	Values
a (Å)	5.589
b (Å)	5.575
c (Å)	5.659
alpha (°)	60.334
beta (°)	119.928
gamma (°)	119.768
V (Å^3)	124.639455
size (Å)	330.581
strain	3.7689 × 10^−5^
R_p_ (%)	10.258
R_wp_ (%)	13.488

**Table 2:** A comparison between reported piezodynamic materials and our work.

Material	Frequency and Power of Ultrasound	Bacterial Type	Time of Ablation	Degradation Efficacy	Year	Reference
Ag@LiNbO_3_/PVDF	40 kHz, 70 W	*S. aureus*	180	96.6	2021	[37]
Ag@LiNbO_3_/PVDF	40 kHz, 70 W	*E. coli*	180	99	2021	[37]
Cu_2_MgSnS_4_/BaTiO_3_	40 kHz	*E. coli* and *S. aureus*	180	>90	2021	[38]
BTO	-	*S. aureus*	-	97.12	2022	[39]
BaTiO_3_	8 Hz	*E. coli*	30	99	2019	[40]
WS_2_ on sapphire substrate	40 kHz	*E. coli*	120	99	2022	[41]
MWCNT-kaolinite-PVDF	33 kHz, 50 W	*E. coli*	45	99	2022	[16]
BFO	15 kHz, 250 W	*E. coli*	60	94	2022	[21]
BF@PV5	10 kHz, 20 W	*E. coli*	30	99.7	This work	This work
BF@PV5	10 kHz, 20 W	*E. faecalis*	30	99.9	This work	This work

## Data Availability

The data will made available on request.
